# KPNA5 Suppresses Malignant Progression of Ovarian Cancer Through Importing the PTPN4 Into the Nucleus

**DOI:** 10.1002/cam4.70731

**Published:** 2025-03-27

**Authors:** Yanming Hu, Jing Zhou, Xinru Ling, Kun Zhao, Peng Huang, Max Gao, Xiaoqing Li, Ming Sun, Yanfen Zou, Guannan Feng

**Affiliations:** ^1^ Suzhou Cancer Center Core Laboratory The Affiliated Suzhou Hospital of Nanjing Medical University, Suzhou Municipal Hospital, Gusu School Suzhou China; ^2^ Department of Oncology The Affiliated Taizhou People's Hospital of Nanjing Medical University Taizhou Jiangsu China; ^3^ Department of Obstetrics and Gynecology The Affiliated Suzhou Hospital of Nanjing Medical University, Suzhou Municipal Hospital, Gusu School Suzhou China; ^4^ Department of Oncology Huaian Hospital of Huaian City Huaian Jiangsu China; ^5^ Department of Computer Science and Engineering University of California Davis California USA; ^6^ Department of Obstetrics and Gynecology The Affiliated Yantai Yuhuangding Hospital of Qingdao University Yantai China

**Keywords:** KPNA5, ovarian cancer, PTPN4, STAT3, tumor suppressor

## Abstract

**Background:**

Abnormal protein localization due to disrupted nucleoplasmic transport is common in tumor cells, but its mechanisms are not well understood. Nuclear pore complexes and nuclear transporter proteins are crucial for protein transport between the nucleus and cytoplasm. Evidence increasingly shows that abnormal expression of karyopherin family proteins disrupts protein translocation, affecting processes like cell differentiation, proliferation, apoptosis, and transcriptional regulation. However, their functions and roles in ovarian cancer remain unclear.

**Methods:**

The expression level of KPNA5 in ovarian cancer tissues and cells was detected by IHC, Western blot, and qPCR. CCK‐8 and colony formation assays were used to assess cell proliferation ability. Transwell assay was conducted to determine cell migration and invasion capacity. A xenograft model was used to assess the effect of KPNA5 on tumor growth in vivo.

**Results:**

KPNA5 expression is downregulated in ovarian cancer (OC) tissues. Low KPNA5 levels were associated with poor survival in OC patients, validated by an OC tissue sample cohort. Overexpression of KPNA5 significantly suppressed OC cell proliferation, tumor growth, and invasion in both in vitro and in vivo studies. Mechanistically, KPNA5 recognizes nuclear localization signals (NLSs) in PTPN4, mediating its nuclear transport and inhibiting STAT3 phosphorylation and its downstream signaling pathway. Similarly, PTPN4 overexpression reduced OC cell viability and invasion, also suppressing STAT3 phosphorylation.

**Conclusions:**

Our findings identify KPNA5 as a tumor suppressor in OC, presenting a potential therapeutic target for OC treatment.

**Abbreviations**
NLSs: nuclear localization signals
NPM: survivin, nucleophosmin
NTRs: nuclear transporters
OC: ovarian cancer
PFS: progression‐free survival
PTPN4: PTP non‐receptor type 4
PY‐STAT1: pyrosine‐phosphorylated STAT1

## 1 Introduction

Ovarian cancer (OC) is among the most common malignancies affecting the female reproductive system, ranking third in incidence after cervical and endometrial cancers. In 2020, there were approximately 314,000 new cases and 207,000 deaths globally, making OC the second deadliest gynecological tumor [1, 2]. Early‐stage OC often presents with no obvious symptoms, leading to about 75% of diagnoses occurring at advanced stages. While surgery and chemotherapy can cure most early‐stage patients, they often fail in advanced stages. Additionally, the recurrence rate within 3 years of initial treatment is as high as 70% [3, 4]. Although the therapeutic options for OC have drastically improved with the rapid development of medicine, for example, PARP inhibitors significantly prolonged the progression‐free survival (PFS) of patients with OC [5, 6]. However, the 5‐year survival rate for advanced ovarian cancer remains unsatisfactory with approximately 30% [7]. Therefore, it is crucial to deepen our understanding of the molecular mechanisms driving OC carcinogenesis and progression to identify new molecular markers for treatment and early diagnosis.

In eukaryotic cells, nuclear import and export are tightly controlled by nuclear transporter proteins. Generally, smaller molecules can enter and leave the nucleus by simple diffusion through the nuclear pore complex; however, larger proteins, RNAs, and other biological units require specialized carrier‐mediated energy‐dependent active transport with the help of the GTPase Ran [8, 9, 10]. Most of the proteins with specialized functions in nuclear transport are members of the nuclear transporter protein family. The two major subfamilies are the import proteins and the export proteins. So far, more than 10 different import proteins and seven different export proteins have been identified [11, 12]. Cancer cells, with their higher rates of proliferation and metabolism, are particularly susceptible to disruptions in nuclear translocation, making these proteins important therapeutic targets [13]. Numerous studies have shown that abnormal expression of nuclear transporter proteins is closely linked to cancer occurrence, progression, and chemotherapy resistance [14, 15, 16, 17, 18]. For instance, the export protein XPO1 is overexpressed in various cancers and is associated with disease progression, treatment resistance, and poorer survival by promoting the cytoplasmic localization of regulatory proteins such as the tumor suppressor p53, CDK1, APC, BRCA1, BRCA2, survivin, nucleophosmin (NPM), and members of the forkhead box family of transcription factors [8, 19]. Additionally, the XPO1 inhibitor Selinexor has been approved by the FDA for the treatment of relapsed refractory myeloma.

KPNA5 belongs to the nuclear transporters (NTRs) karyopherin alpha (KPNA) family, which also contains KPNA1, KPNA2, KPNA3, KPNA4, KPNA6, and KPNA7. On the basis of sequence homology, the KPNA family can be categorized into three subfamilies: α1, α2, and α3. KPNA5, together with KPNA1 and KPNA6, belongs to the α1 subfamily [20, 21]. Generally, KPNA proteins exert their nucleocytoplasmic transport function by acting as adaptor molecules with Karyopherin beta (KPNB) to carry protein cargoes with NLS from the cytoplasm to the nucleus. Besides, KPNA also possesses non‐transport properties such as spindle assembly, nuclear membrane formation, cytoplasmic retention, protein degradation, and gene expression regulation function. A previous study reveals that the Ebola virus VP24 protein (eVP24) can bind to KPNA5, thereby inhibiting PY‐STAT1 nuclear translocation and rendering cells resistant to IFNs [22]. Besides, a recent study indicates that KPNA5 is highly expressed in pancreatic cancer patients without gemcitabine resistance, and upregulation of KPNA5 increases the response of gemcitabine‐resistant PANC‐1 cells to gemcitabine [23].

The PTP non‐receptor type 4 (PTPN4) is identified as an important regulator in cerebellar synaptic plasticity. Recently, a growing number of evidence have demonstrated that PTPN4 expression is downregulated in multiple types of cancers, which indicates that PTPN4 may function as a tumor suppressor. For example, Fu et al. found that PTPN4 was lowly expressed in patients with relapse breast cancer, and patients with a high level of PTPN4 had a longer overall survival and relapse‐free survival [24]. In addition, PTPN4 could suppress rectal cancer cell growth by dephosphorylating pSTAT3 at the Tyr705 residue, thereby impairing the transcriptional activity of STAT3 [25]. However, to date, the roles of KPNA5 and PTPN4 in the tumorigenesis and progression of OC remain poorly understood. In this study, we found that KPNA5 expression is downregulated in OC through comprehensive analysis and explored its function as a tumor suppressor and underlying molecular regulatory mechanism.

## 2 Materials and Methods

### 2.1 Bioinformatics Analysis

All protein coding genes belong to the karyopherin family were collected from NCBI database and followed by manually double check. The location distribution of all 25 KYPs were visualized via the karyoplot R package. Afterward, transcriptomic datasets of ovarian cancer tissues and normal ovary samples were collected from GEO database. Specifically, three GEO microarray‐based datasets (GSE29450 [26], GSE10971 [27], and GSE6008 [28]) were collected for differential expression analyses. Gene expression and associated clinical metadata were collected via the GEOquery package, and optional probe to gene annotation was fetched by utilizing the idmap function of the AnnoProbe package. Differential expression analyses were performed via limma‐voom with default parameters. Only probes with FDR (Benjamini & Hochberg) lower than 5% were identified as significant differential expressed gene probes. For simplicity, genes with multiple significant probes were pruned to keep only the most significant one with the lowest adjusted *p*‐value. Identified significantly activated or repressed KYPs were visualized via the ComplexHeatmap package. Consistent upregulated and downregulated KYPs were visualized via the SuperExactTest package. For replication, the aberrant repression of KPNA5 were further confirmed in RNA‐seq based large OC cohorts: TCGA‐OV (versus normal ovary tissue samples from GTEx using recomputed gene expression from consistent reanalyzed TCGA and GTEx samples by UCSC Toil RNAseq Recompute Compendium) and GSE209964 [29] via Wilcox test. The associated between KPNA5 expression and tumor stage were examined via Wilcox test in TCGA‐OV and CPTAC2‐Ovary cohort, whose gene expression and clinical data were collected from GDC database. Survival analysis were performed and visualized via the survminer package. Specifically, the optimal cutpoint for KPNA5 expression were determined via the surv‐cut point function and log‐rank test were employed to examine the significant difference between KPNA5‐high and KPNA5‐low patient groups.

### 2.2 Cell Culture

The HEK‐293T human embryonic kidney epithelial cell line and the ovarian cancer cell lines SKOV‐3 and OVCAR‐3 were procured from the National Collection of Authenticated Cell Cultures located in Shanghai, China. Specifically, SKOV‐3 cells were cultured using McCoy's 5A Medium (Thermo Fisher Scientific, MA, USA). HEK‐293T and OVCAR‐3 cell lines were maintained in DMEM medium (Thermo Fisher Scientific, MA, USA). All culture media were supplemented with 10% fetal bovine serum (Thermo Fisher Scientific, MA, USA). The cells were cultivated in a humidified incubator under conditions of 5% CO_2_ at 37°C, and routine monthly testing for mycoplasma contamination was performed.

### 2.3 Plasmid Construction and siRNA Transfection

The expression plasmid for KPNA5 was synthesized and cloned using the pLVX‐Blast vector; PTPN4‐WT and PTPN4‐Mut sequences were synthesized and cloned into the pLVX‐puro vector. The specific siRNAs for KPNA5 were synthesized by Ri‐bobio (Ribobio, Guangzhou, China). They were transfected into ovarian cancer cells using Lipofectamine 3000 reagent (Thermo Fisher Scientific, MA, USA) according to the manufacturer's protocol. The sequence of siRNAs is shown in Table S1. Total RNA and protein were collected 48 h after transfection. The qPCR and Western blot methods were performed to examine overexpression and knockdown efficiency.

### 2.4 Construction of Cell Lines With Stable Expression

For lentivirus packaging, pLenti‐CMV‐Flag‐Blast (Addgene, MA, USA) harboring the target genes cDNA sequence was co‐transfected with psPAX2 (Addgene, MA, USA) and PMD2.G (Addgene, MA, USA) at 4:3:2 ratios into HEK‐293 T cells. 48 h later, supernatant with lentivirus was collected and filtrated through a 0.45 μm filter to remove the cell debris. Lentivirus expressing the indicated gene was used to infect SKOV‐3 and OVCAR‐3 cells. Two days after infection, blasticidin S (Invitrogen, CA, USA) was added at a final concentration of 2 μg/mL to select the stably expressed cells.

### 2.5 Western Blotting

Cells were suspended in RIPA buffer with freshly added 1X protease inhibitor. After 20 min on ice, lysates were clarified by centrifugation for 15 min at 12,000 g at 4°C. Protein concentration was measured in clarified lysates using a BCA Protein Assay kit (Beyotime, Shanghai, China), and proteins were resolved by SDS‐PAGE before being transferred to 0.45 μm PVDF films (MilliporeSigma, MA, USA) using transfer buffer. The films were blocked in 5% milk and hybridized overnight at 4°C with the following primary antibodies: anti‐KPNA5 (1:1000 dilution), anti‐PTPN4 (1:1000 dilution), anti‐STAT3 (1:1000 dilution), anti‐p‐STAT3 (1:1000 dilution), anti‐Flag (1:2000 dilution), anti‐GFP (1:1000 dilution), anti‐H3 (1:2000 dilution), and anti‐GAPDH (1:2000 dilution). The immunocomplexes were subsequently incubated with the HRP‐conjugated secondary antibodies, and chemiluminescence detection was performed using ECL followed by imaging on a BioRad Versadoc. The signal intensity of the bands was analyzed by using the ImageJ program. The information of the primary and secondary antibodies used in western blots is listed in Table S1.

### 2.6 Immunofluorescence Staining

Cells inoculated onto cell slides in 48‐well plates were fixed with 4% paraformaldehyde for 10 min and then incubated with 0.1% Triton X‐100 for 10 min. After being blocked with 5% BSA for 30 min, the primary antibody was incubated at 4°C overnight. After incubation with the corresponding fluorescent secondary antibody for 1 h the following day, cell nuclei were stained using DAPI after adding Antifade Mounting Medium. Images were taken using an Olympus (FV1200) confocal microscope. And the images were processed using FV10‐ASW_View4.

### 2.7 Cell Viability Assay and Colony Formation Assay

SKOV‐3 and OVCAR‐3 cells were transfected with siRNAs and collected (48 h post transfection) and inoculated into 96‐well plates at a concentration of 1000 cells per well, and 10 μL CCK‐8 solution (Beyotime, Shanghai, China) was added to each well at 0 h, 24 h, 48 h, 72 h, and 96 h. The timing was started after the cells were attached to the well. The cells were incubated at 37°C for 2 h. The absorbance at 450 nm was measured with a microplate reader. For the colony formation assay, 1000 or 400 cells (for siRNA transfected cells) were seeded into a 6‐well plate, with three replicate wells for each cell. After growing for 14 days, they were fixed with 4% paraformaldehyde and stained with crystal violet. ImageJ was used to count colonies for statistical analysis.

### 2.8 Transwell Assay

To determine the migration and invasion of cells, 5 × 10^4^ cells or 2 × 10^4^ cells (for siRNA transfected cells) were plated in medium without serum in the top chamber of a transwell with an 8.0 μm pore size (Corning, NY, USA). For invasion assays, the transwell membranes were pre‐coated with Matrigel (1:4 dilution) (BD Biosciences, USA). The bottom chamber contained standard medium with 20% FBS medium. After being cultured for 48 h, the cells were fixed with 4% paraformaldehyde and stained with crystal violet (Solar‐bio Technology, Beijing, China).

### 2.9 Co‐IP and MS

Cells were lysed in IP lysate with added protease inhibitors for 2 h at 4°C, centrifuged, and the supernatant was saved for direct Western blotting. For co‐immunoprecipitation, protein lysates were incubated with microbead‐coupled anti‐Flag or anti‐GFP overnight at 4°C in a rotary shaker and then incubated with Protein A/G Magnetic Beads (Med Chem Express, NJ, USA) for 2 h. The immunocomplexes were then washed 5 times with IP lysis buffer, then separated by SDS‐PAGE and analyzed by Western blotting. To explore the cargo proteins of KPNA5, we generated KPNA5‐Flag stably expressed SKOV3 cells. Subsequently, immunoprecipitation was conducted with a Flag antibody, and enriched proteins were eluted for subsequent mass spectrometer analysis using a Q‐Exactive mass spectrometer (Thermo Fisher Scientific, MA, USA) equipped with a nano‐electrospray ion source. Protein identification was executed through the Mascot program (v2.3.02) and compared against the Uniprot Human Protein Database with default parameters (false discovery rate ≤ 1% and score *R* ≥ 40).

### 2.10 IHC

The tumor tissue underwent fixation in 10% formalin and subsequent paraffin embedding. Immunostaining procedures were conducted for H&E, Ki67 (1:100 dilution) and KPNA5 (1:50 dilution) employing diaminobenzidine as the chromogenic substrate to amplify and visualize the immunoreactivity signal. Counterstaining with hematoxylin was employed to enhance tissue contrast. Image acquisition was performed utilizing an inverted confocal laser scanning microscope.

### 2.11 Animal Experiment

4‐week‐old female BALB/c mice were procured from Gempharmatech for the purpose of this study. To establish the subcutaneous tumor transplantation model, SKOV‐3 cells from different treatment groups were suspended in PBS and subsequently inoculated subcutaneously into BALB/c mice. Tumor volume was calculated using the formula: long diameter × short diameter^2/2, which was measured weekly throughout the experimental period. Following a duration of 7 weeks, euthanasia was performed on the mice, tumors were excised and weighed, and subsequent histological analyses including H&E staining and IHC staining were conducted. The study was approved by the Institutional Ethics Committee of Nanjing Medical University (IACUC2103036).

### 2.12 Statistical Analysis

Data analysis was performed using GraphPad Prism 8.0 software packages. Statistical differences between two groups of data were assessed using two‐tailed Student's *t*‐tests, while One‐way ANOVA with Tukey's post hoc test was employed to examine statistical differences across multiple groups. A significance level of *p* < 0.05 was considered statistically significant in this study.

## 3 Results

### 3.1 Progressive Inactivation and Poor Survival Association of Ovary‐Enriched KPNA5 in Ovarian Cancer

**FIGURE 1:**
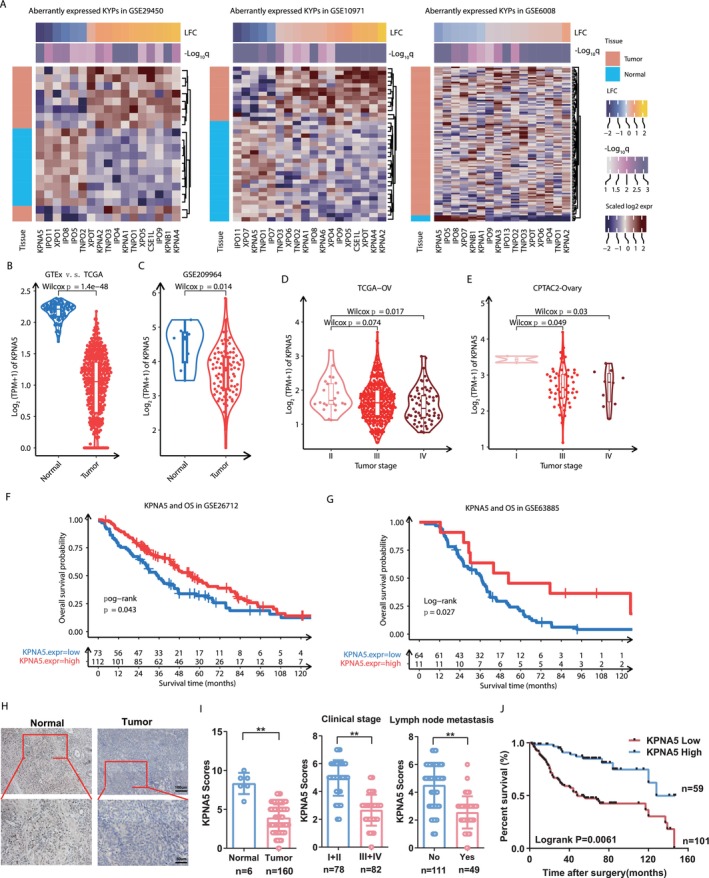
Aberrantly repressed expression of KPNA5 and its positive association with prognostic outcomes in ovarian cancer. (A) Heatmap of significantly differential expressed karyopherin family member genes (KYPs) in ovarian tumor tissue samples versus normal ovary tissue samples from three microarray‐based GEO cohorts. (B, C) Replication of significant downregulation of KPNA5 in OC tumor samples from large RNA‐seq based OC cohorts: TCGA and GSE209964. (D, E) Negative association of KPNA5 expression and tumor stage in TCGA‐OV (D) and CPTAC2‐Ovary (E) cohort. (F, G) Higher expression of KPNA5 were associated with longer overall survival period in GSE26712 (F) and GSE63885 (G). (H) IHC staining analysis of KPNA5 protein expression levels in ovarian cancer tissues and normal ovarian tissues. (I) Correlation analysis the association between KPNA5 expression and patients' clinical stage and lymph node metastasis. (J) Lower expression level of KPNA5 is associated with shorter overall survival time of ovarian cancer patients in our cohort.

We collected all gene members belonging to the Karyopherin family from the NCBI database and obtained a total of 25 protein‐coding genes, which were universally distributed among all chromosomes (Figure S1A). Among these 25 KYPs, differential expression analyses identified 17 (6 down and 11 up) differentially expressed genes (DEGs) in GSE29450, 18 (five down and 13 up) DEGs in GSE10971, and 16 (6 down and 10 up) DEGs in GSE6008 (Figure 1A), which revealed extensive aberrant expression of KYPs in ovarian cancer. Note of worth, four genes (IPO9, KPNA2, TNPO3, and XPOT) and one gene (KPNA5) (Figure S1B) were consistently significantly up‐ and down‐regulated in ovarian cancer, respectively. According to the rank of fold change in tumor tissue and the tissue‐specific expression pattern in human normal tissues, KPNA5 was highlighted since it displayed the most severe downregulation in two of the above three datasets (GSE29450 and GSE6008) and the highest ovarian specificity among all KYPs, which was independent of age and gender (Figure S1C–E). Hence, KPNA5 was picked out for further investigation. Transcriptional repression of KPNA5 observed in the above microarray‐based datasets was successfully replicated in two large OC cohorts including TCGA (Figure 1B) and GSE209964 (Figure 1C). Meanwhile, KPNA5 displayed significantly lower expression during stage III and stage IV when compared with early‐stage OC patients (Figure 1D,E). Furthermore, survival time association tests suggested OC patients with higher KPNA5 expression were significantly associated with superior overall survival time (Figure 1F,G).

### 3.2 KPNA5 Suppresses Cell Proliferation and Tumor Growth

**FIGURE 2:**
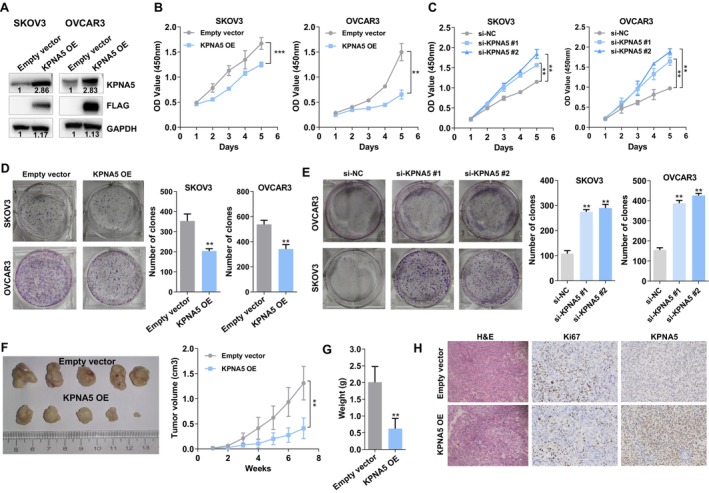
KPNA5 exerts tumor suppressive roles in ovarian cancer cells. (A) Western blot analysis was conducted to determine the overexpression efficiency of KPNA5 in SKOV3 and OVCAR3 cells. (B) CCK8 assay was conducted to determine the effect of KPNA5 overexpression on the cell proliferation of SKOV3 and OVCAR3 cells. (C) CCK8 assay was conducted to determine the effect of KPNA5 downregulation on the cell proliferation of SKOV3 and OVCAR3 cells. (D) colony formation assay was conducted to determine the effect of KPNA5 overexpression on the colony‐forming capacity of SKOV3 and OVCAR3 cells. (E) colony formation assay was conducted to determine the effect of KPNA5 downregulation on the colony‐forming capacity of SKOV3 and OVCAR3 cells. (F) Subcutaneous xenograft tumor model was generated to determine the effect of KPNA5 overexpression on SKOV3 cells derived tumor growth in vivo. (G) Weighing of the tumors from mice. (H) H&E and IHC staining analysis of the Ki67 and KPNA5 expression levels in the tumor tissues from the mice. Three individual experiments were performed, and error bars of the data indicate mean ± SEM, **p < 0.01; ***p < 0.001.

To confirm the altered expression of KPNA5 in ovarian cancer, we examined the protein level of KPNA5 in ovarian cancer tissues using IHC staining. The results showed that KPNA5 expression was lower in ovarian cancer tissues compared to adjacent normal tissues (Figure 1H). Additionally, low KPNA5 expression was significantly associated with advanced clinical stages and lymph node metastasis (Figure 1I). Kaplan–Meier survival analysis revealed that ovarian cancer patients with low KPNA5 expression had shorter overall survival times (Figure 1J). To further investigate the functionality of KPNA5 in ovarian cancer cells, KPNA5 expression was upregulated by transduction with lentivirus containing the KPNA5 cDNA or downregulated via transfection with siRNAs in SKOV3 and OVCAR3 cells. The overexpression and knockdown efficiency were validated by Western blot (Figure 2A, Figure S1F). CCK‐8 and colony formation assays showed that KPNA5 overexpression reduced the proliferation and clonogenicity of SKOV3 and OVCAR3 cells, while KPNA5 knockdown had the opposite effect (Figure 2B–E). Moreover, the results of the subcutaneous xenograft model showed that overexpression of KPNA5 significantly repressed the tumor growth (Figure 2F), and KPNA5 overexpressed SKOV3 cells derived tumors displayed lighter weights and lower Ki67 expression levels compared with control (Figure 2G,H).

### 3.3 KPNA5 Inhibits Ovarian Cancer Cell Migration and Invasion In Vitro

**FIGURE 3:**
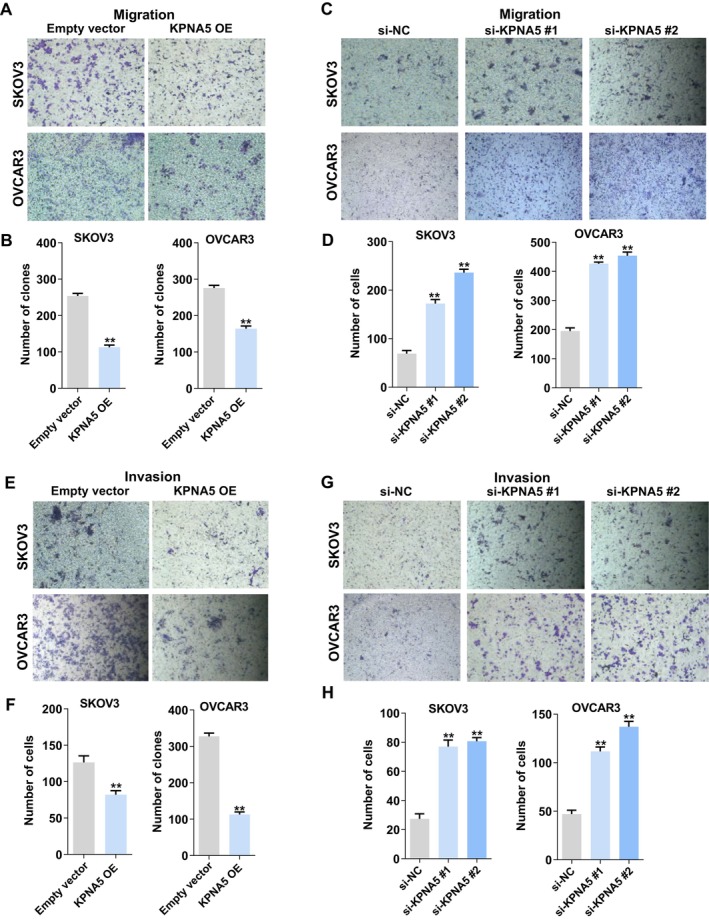
KPNA5 represses ovarian cancer cells migration and invasion in vitro. (A, B) Transwell assays were conducted to determine the effect of KPNA5 overexpression on SKOV3 and OVCAR3 cells migration. (C, D) Transwell assays were conducted to determine the effect of KPNA5 downregulation on SKOV3 and OVCAR3 cells migration. (E, F) Transwell assays were conducted to determine the effect of KPNA5 overexpression on SKOV3 and OVCAR3 cells invasion. (G, H) Transwell assays were conducted to determine the effect of KPNA5 downregulation on SKOV3 and OVCAR3 cells invasion. Three individual experiments were performed, and error bars of the data indicate mean ± SEM, **p < 0.01.

Since the expression level of KPNA5 correlated with lymph node metastasis in patients, we further explored the effect of KPNA5 on the migration and invasive ability of ovarian cancer cells. The results of transwell assays indicated that overexpression of KPNA5 significantly impaired SKOV3 and OVCAR3 cells migration capacity, while knockdown of KPNA5 expression promoted the ability of SKOV3 and OVCAR3 cells migration (Figure 3A–D). Consistently, overexpression of KPNA5 also remarkably inhibited SKOV3 and OVCAR3 cells invasive capacity, while knockdown of KPNA5 expression resulted in the enhanced invasion of SKOV3 and OVCAR3 cells (Figure 3E–H).

### 3.4 KPNA5 Promotes the Nucleus Transport of PTPN4 Protein by Recognizing NLS

**FIGURE 4:**
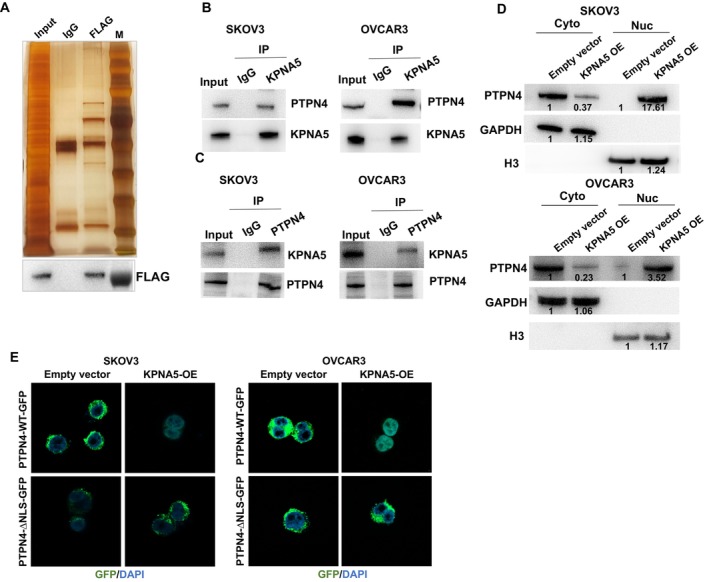
KPNA5 mediates the nuclear transporting of cargo protein PTPN4. (A) Silver‐stained image shows the protein bands interacting with KPNA5 in SKOV3 cells. (B, C) IP and Western blot analysis of the interaction between KPNA5 and PTPN4 in ovarian cancer cells. (D) Nucleus and cytoplasm fraction analysis of the effect of KPNA5 on PTPN4 nucleus translocation. (E) IF staining assays determine the effect of KPNA5 overexpression on PTPN4 protein distribution in SKOV3 and OVCAR3 cells. Three individual experiments were performed, and error bars of the data indicate mean ± SEM.

To further explore the underlying mechanism that KPNA5 exerts its tumor‐suppressive function, we identified the potential cargo proteins that bind with KPNA5 by using immunoprecipitation and mass spectrometry analysis (IP‐MS). The silver staining results revealed a lot of proteins that bind with KPNA5 in ovarian cancer cells (Figure 4A). Among the many proteins that interacted with KPNA5 (Table S2), PTPN4 is the one that attracted our interest. Recent studies have indicated that PTPN4 was able to suppress the transcription activities of STAT3 by impairing its phosphorylation, while inhibition of PTPN4 activated phosphorylation of STAT3 [30]. As we know, STAT3 is a well‐known transcription factor with cancer‐promoting function. Hence, PTPN4 was chosen as an important cargo protein candidate of KPNA5. The interaction between KPNA5 and PTPN4 was further confirmed by co‐IP and Western blot in ovarian cancer cells (Figure 4B,C). Moreover, overexpression of KPNA5 could significantly promote the transport of PTPN4 from cytoplasm to nucleus in ovarian cancer cells (Figure 4D). KPNA transporter proteins mediate nuclear transport of cargo proteins mainly by recognizing classical or non‐classical nuclear localization sequences (NLS). Subsequently, we identified a non‐classical NLS in the context of the PTPN4 protein sequence (79–102 aa) by using an online bioinformatics analysis tool. According to the prediction results, we constructed a wild‐type and mutant (without NLS) PTPN4 expression vector. The results of IF staining showed that deletion of the NLS sequence impeded the nuclear transport of the PTPN4 protein by KPNA5. These data demonstrated that PTPN4 might be an important cargo protein of KPNA5 in ovarian cancer cells.

### 3.5 PTPN4 Exerts Tumor Suppressive Function via Regulating Phosphorylation STAT3 Axis

**FIGURE 5:**
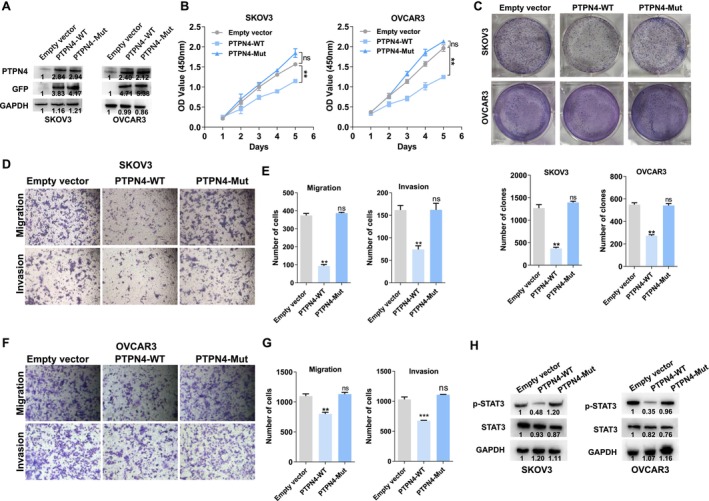
KPNA5 exerts tumor suppressive function through regulating PTPN4/STAT3 signaling pathway. (A) Western blot was conducted to determine the overexpression efficiency of wide‐type and mutant PTPN4 in ovarian cancer cells. (B) CCK8 assays were performed to evaluate the effect of PTPN4‐WT and PTPN4‐Mut expression on ovarian cancer cells proliferation. (C) Colony formation were performed to evaluate the effect of PTPN4‐WT and PTPN4‐Mut expression on ovarian cancer cells clone formation capacity. (D–G) Transwell assays were performed to evaluate the effect of PTPN4‐WT and PTPN4‐Mut expression on ovarian cancer cells migration and invasion. (H), Western blot was conducted to determine the overexpression efficiency of wide‐type and mutant PTPN4 on the STAT3 protein phosphorylation. Three individual experiments were performed, and error bars of the data indicate mean ± SEM, **p < 0.01; **p < 0.001; ns, no significant.

Although a few studies have suggested that PTPN4 could function as a tumor suppressor gene, its role in ovarian cancer is unclear. To further investigate the function of PTPN4 in ovarian cancer, the SKOV3 and OVCAR3 cells were transfected with an empty vector, PTPN4‐WT, or PTPN4‐Mut vector (without NLS) and the efficiency was determined by Western blot (Figure 5A). The CCK‐8 and clone formation experiments showed that overexpression of wild‐type PTPN4 could inhibit the proliferation and clonegenesis ability of SKOV‐3 and OVCAR‐3 cells; however, the repressive effects were abolished when depleted the NLS from the PTPN4 protein sequence (Figure 5B,C). Consistently, the results of transwell assays indicated that wild‐type PTPN4, but not the mutant PTPN4, could inhibit migration and invasive ability of SKOV‐3 and OVCAR‐3 cells (Figure 5D–G). Furthermore, Western blot analysis indicated that overexpressing wild‐type, not the mutant PTPN4, diminished the expression of p‐STAT3 (Figure 5H). The aforementioned results suggested that PTPN4 also exerts a tumor suppressive function in ovarian cancer by influencing the phosphorylation of STAT3, which is dependent on the NLS.

## 4 Discussion

In our study, we discovered that KPNA5 was abundantly expressed in normal ovarian tissues but significantly downregulated in ovarian cancer tissues. Low KPNA5 expression was associated with a poor prognosis in ovarian cancer patients. Furthermore, exogenous overexpression of KPNA5 significantly inhibited the proliferation and invasion of ovarian cancer cells in vitro, as well as the growth of xenograft tumors in vivo. These findings suggest that KPNA5 functions as a tumor suppressor in ovarian cancer cells. However, it remains unclear which cargo proteins KPNA5 mediates into the nucleus to exert its tumor‐suppressive effects.

KPNA5 belongs to an important member of the importin family, which is mainly responsible for the transport of large molecular weight cargo proteins from the cytoplasm to the nucleus to exert their biological function. For example, KPNA5 could recognize the NLS and mediate nuclear transport of pyrosine‐phosphorylated STAT1 (PY‐STAT1), while ebola virus VP24 protein would compete with PY‐STAT1 to bind KPNA5, resulting in the impaired PY‐STAT1 nuclear transport [31]. As well as our findings, Zhou et al. also found that KPNA5 could exert tumor suppressive function in pancreatic cancer [32]; however, its cargo protein was not characterized. In this study, we found that PTPN4 is a key cargo protein of KPNA5 in ovarian cancer cells by mass spectrometry analysis of KPNA5‐bound proteins. Bioinformatics analysis identified a NLS sequence in the context of PTPN4 protein, and deletion of this NLS sequence significantly inhibited KPNA5‐mediated transport of PTPN4 into the nuclear. These findings suggest that KPNA5 may function as a tumor suppressor by partially mediating the nuclear transport of PTPN4 in ovarian cancer cells.

PTPN4, a member of the Class I non‐receptor protein phosphatases, contains a single PTP domain, catalytic domains in the C‐terminal, PDZ and FERM domains outside of the catalytic domain [33]. Emerging evidence has revealed that wildly expressed PTPN4 is involved in various biological processes. PTPN4 affects the innate immune responses through dephosphorylating the immunoreceptor tyrosine‐based activation motif in the TCR ξ subunit and repressing the activation of AP‐1 and NF‐κB [34]. Recently, several studies have found that PTPN4 can function as a tumor suppressor in various cancers. For instance, elevated miR‐375 promotes cell proliferation, invasion, and induces enzalutamide resistance in prostate cancer cells by targeting PTPN4, which in turn enhancing the phosphorylation of STAT3 [30]. Besides, MARCH8 facilitated the proliferation and metastasis of pancreatic cancer by inducing the degradation of PTPN4 protein and thereby activating the STAT3 phosphorylation [35]. Additionally, PTPN4 suppresses the growth of rectal cancer cells through dephosphorylating pSTAT3 at the Tyr705 residue [25]. Here, we found that the expression of wild‐type PTPN4 significantly inhibited the proliferation and invasive ability of ovarian cancer cells, but mutant PTPN4 with the deletion of the NLS sequence failed to induce significant tumor suppression. In addition, along with other studies, we also found that PTPN4 functions as a tumor suppressor in OC mainly by inhibiting the phosphorylation of STAT3, which may further hinder the activation of the subsequent pathway and inhibit ovarian cancer tumor growth and metastasis.

## 5 Conclusions

In summary, our findings reveal for the first time that KPNA5 expression is downregulated in ovarian cancer and is associated with patients' poor prognosis. KPNA5 functions as a tumor suppressor gene by mediating the nuclear transporting of the cargo protein PTPN4, which in turn inhibits STAT3 phosphorylation and downstream pathway activation. However, there are some limitations of this study. For example, the link between KPNA5, PTPN4, and STAT3 is not fully defined, which needs to be further investigated. The present study may provide new perspectives in understanding the development and progression of ovarian cancer.

## Author Contributions

M.S., G.F., and Y.Z. designed and supervised the study. Y.H., X.L., and J.Z. conducted the experiment. K.Z., and P.H. contributed to the acquisition of results. Y.H., A.G., and X.L. performed the data analysis. Y.Z., and G.F. wrote the manuscript. All the authors read and approved the final manuscript.

## Ethics Statement

The animal study protocol was approved by the Institutional Ethics Committee of Nanjing Medical University (IACUC2103036).

## Conflicts of Interest

The authors declare no conflicts of interest.

## Supporting information


**Figure S1** Tissue‐enhanced KPNA5 expression in normal ovary. (A) Ideogram plot of 25 karyopherin family member protein coding genes (KYPs) in hg38. (B) Overlap of significant upregulated KYPs and downregulated KYPs in three GEO cohorts. (C) Heatmap of scaled expression of KYPs among 54 normal GTEx tissues. (D) Age‐ and gender‐independent enhanced expression of KPNA5 in ovary when compared with other normal tissues. (E) Boxplot of log‐transformed TPM of KPNA5 among all GTEx samples. (F) Western blot analysis of the KPNA5 siRNA knockdown efficiency. (G) Bioinformatics analysis of the NLS in the context of KPNA5 protein.

**Table S1** Antibody information, sequences of siRNA and primers.

**Table S2** List of IP‐MS identified proteins.

## Data Availability

The dataset(s) supporting the findings of this study is included within the article. Requests for materials and supporting data should be addressed to Prof. Guannan Feng.
