# Supplementary figures and images for: KPNA5 Suppresses Malignant Progression of Ovarian Cancer Through Importing the PTPN4 Into the Nucleus
Source: Cancer Med. 2025 Mar 27;14(7):e70731. doi: 10.1002/cam4.70731 (PMC11947769; doi:10.1002/cam4.70731)

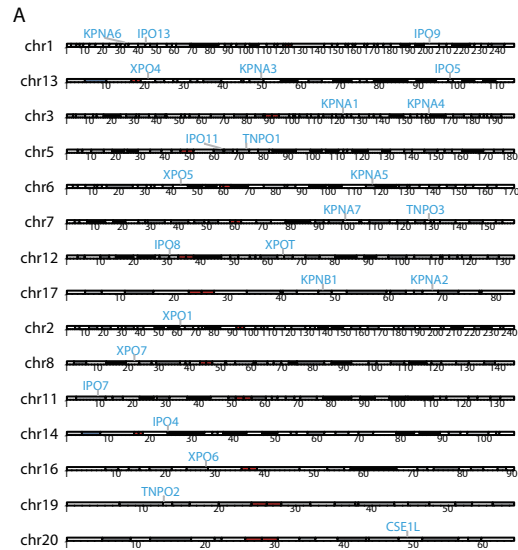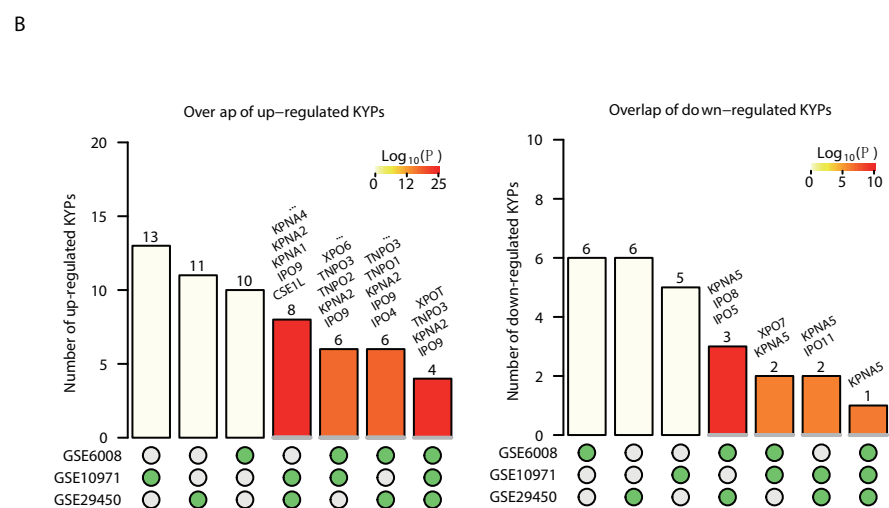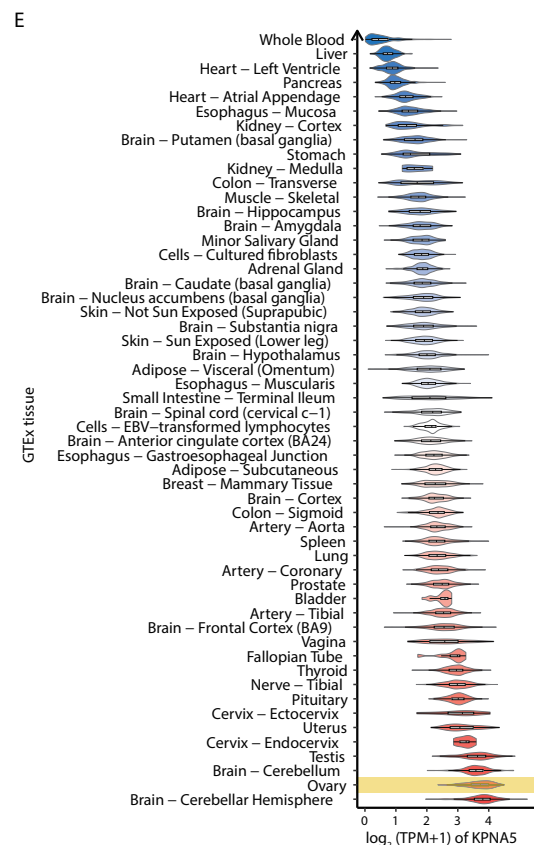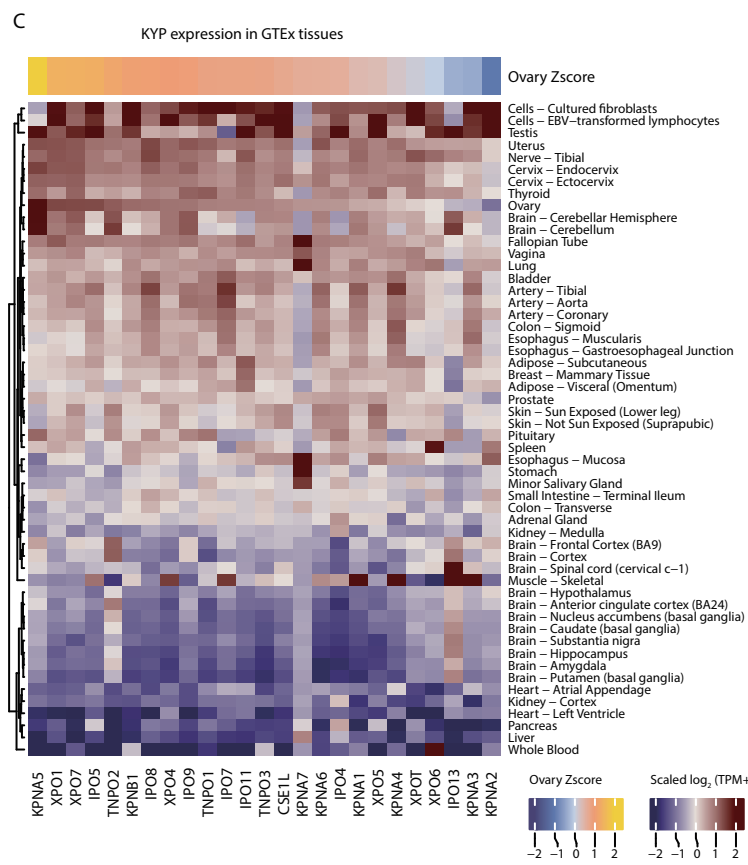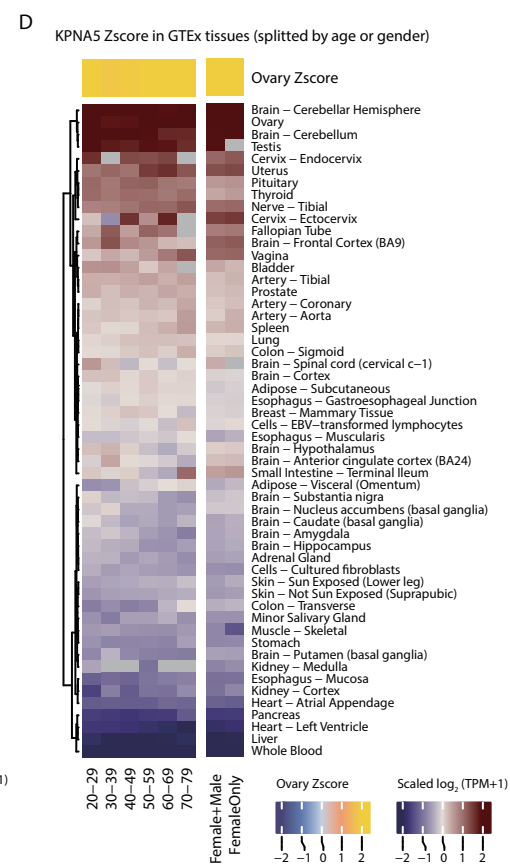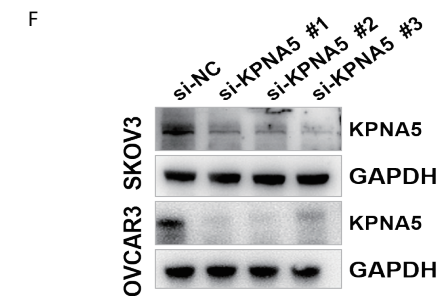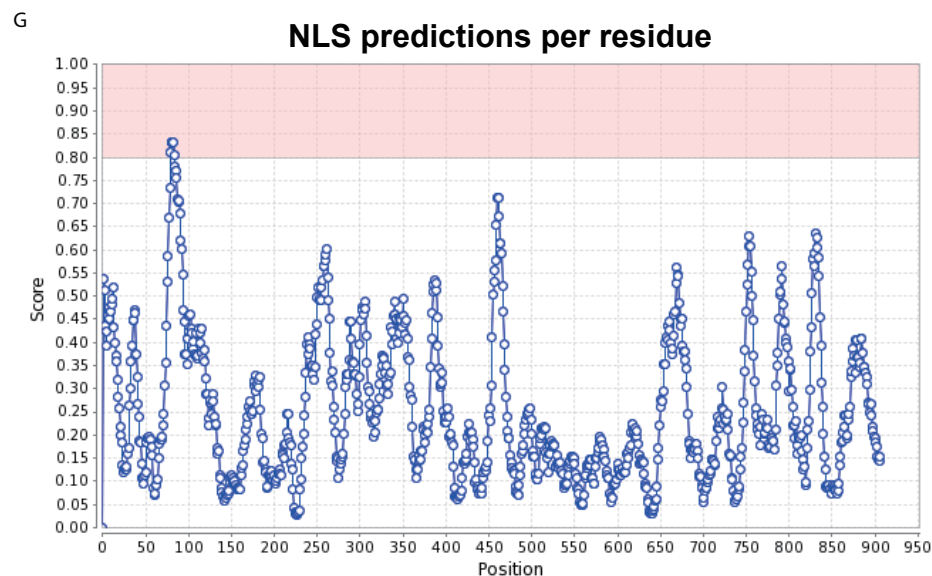

Supplement: Supplementary file 1 — Figure S1 Tissue‐enhanced KPNA5 expression in normal ovary. (A) Ideogram plot of 25 karyopherin family member protein coding genes (KYPs) in hg38. (B) Overlap of significant upregulated KYPs and downregulated KYPs in three GEO cohorts. (C) Heatmap of scaled expression of KYPs among 54 normal GTEx tissues. (D) Age‐ and gender‐independent enhanced expression of KPNA5 in ovary when compared with other normal tissues. (E) Boxplot of log‐transformed TPM of KPNA5 among all GTEx samples. (F) Western blot analysis of the KPNA5 siRNA knockdown efficiency. (G) Bioinformatics analysis of the NLS in the context of KPNA5 protein. [file CAM4-14-e70731-s003.pdf]
